# Microbial Transformations of 7-Methoxyflavanone

**DOI:** 10.3390/molecules171214810

**Published:** 2012-12-11

**Authors:** Edyta Kostrzewa-Susłow, Tomasz Janeczko

**Affiliations:** Department of Chemistry, Wrocław University of Environmental and Life Sciences, Norwida 25, Wrocław 50-375, Poland; E-Mail: janeczko13@interia.pl

**Keywords:** biotransformation, 7-methoxyflavanone, *Aspergillus niger*, *Aspergillus ochraceus*, *Penicillium chermesinum*

## Abstract

Microbial transformations of racemic 7-methoxyflavanone using strains of the genus *Aspergillus *(*A. niger *KB, *A. ochraceus *456) and the strain *Penicillium chermesinum *113 were described. The strain *A. niger *KB catalysed carbonyl group reduction, leading to (±)-2,4-*cis*-7-methoxyflavan-4-ol. Biotransformation with the help of *A. ochraceus *456 gave two products: (+)-2,4-*trans*-7-methoxyflavan-4-ol and 4'-hydroxy-7-methoxyflavone. Transformation by means of *P. chermesinum *113 resulted in a dihydrochalcone product, 4,2'-dihydroxy-4'-methoxydihydrochalcone. DPPH scavenging activity test proved that all the biotransformations products have higher antioxidant activity that the substrate.

## 1. Introduction

Flavonoids are polyphenolic compounds with diverse chemical structures, which are widely found in plants. Apart from plants, a natural capability to carry out biosynthesis of flavonoid compounds is a feature of some endophytic fungi [1]. Animal and human organisms do non synthesize flavonoids [2,3]. Their specific properties make them useful for pharmaceutical, cosmetics and food industry. Therefore, there is growing interest in chemical synthesis of flavonoids, as well as in their biotechnological production [4,5,6,7,8,9]. The therapeutic potential and low toxicity of flavonoids are accompanied with relatively little information about their metabolic pathways in living organisms. That is why the attention has been directed to biocatalysis [10].

The microorganisms used in biotransformation of flavonoid compounds have enzymatic systems capable of performing various chemical reactions, including reduction, hydroxylation, *O*-methylation and hydrolysis [11,12]. Analytical tests carried out during the course of biotransformation allow tracing of the metabolic transformations of flavonoids [13,14,15]. The products are often new compounds, not described in the literature so far and difficult to obtain by chemical synthesis. Moreover, they have often high antioxidant properties [16,17].

Biotransformation of compounds with a methoxy group in the C-7 position of flavanone were described by Ibrahim and co-workers [18]. The strain *Cunninghamella elegans* NRRL 1392 transformed 7-*O*-methylnaringenin (sakuranetin) into naringenin and naringenin-4'-sulfate, and 5,3',4'-trihydroxy-7-methoxyflavanone into eriodictyol-4'-sulfate, whereas biotransformation of 5,4'-dihydroxy-7,3'-dimethoxyflavanone afforded homoeriodictyol (5,7,4'-trihydroxy-3'-methoxyflavanone) and homoeriodictyol-7-sulfate. The observed sulfatation and *O*-demethylation proceeded regio-selectively [18].

The objective of our research was to transform racemic 7-methoxyflavanone into optically pure products with higher antioxidant properties than the starting substrate.

## 2. Results and Discussion

An initial screening of 27 filamentous fungi of the genera *Aspergillus*, *Penicillium*, *Coryneum*, *Nectria*, *Chaetomium*, *Absidia*, *Spicaria*, and *Cryptosporiopsis *[19] allowed us to select three microorganisms (*Aspergillus niger* KB, *Aspergillus ochraceus* 456 and *Penicillium chermesinum* 113) that were capable of transforming racemic 7-methoxyflavanone (**1**).

**Scheme 1:**
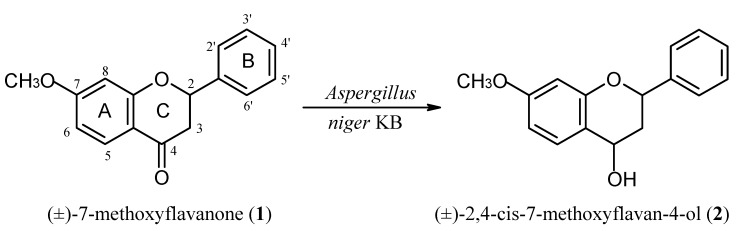
Reduction of the carbonyl group of 7-methoxyflavanone with *A. niger *KB.

The wild strain *A. niger *KB, as in the case of flavanone [20], 6-hydroxyflavanone [20] and 7-hydroxyflavanone [19], performed a reduction of the carbonyl group of 7-methoxyflavanone (**1**) (Scheme 1).

Racemic 7-methoxyflavan-4-ol (**2**) was obtained in 76% yield (76 mg from 100 mg of the substrate) after 9 days of biotransformation. The reaction was continued until the substrate was fully consumed. The reduction of the carbonyl group of 7-methoxyflavanone (**1**) was confirmed in the ^1^H-NMR spectrum of **2** by the presence of a wide singlet of one proton at δ = 5.05 ppm, corresponding to H-4. In the ^13^C-NMR the chemical shift of C-4 is moved from δ = 192.2 ppm for substrate **1** to δ = 65.6 ppm for 7-methoxyflavan-4-ol (**2**). Additionally, in the IR spectrum of **2** a new hydroxyl group absorption band appeared at 3225 cm^−1^. In the ^1^H-NMR spectrum of **2** the chemical shifts of H-2, H-4, H-3_ax_, and H‑3_eq_, as well as the respective coupling constants were compared with the carbonyl group reduction products obtained in transformation of 6- and 7-hydroxyflavanone using the strain *A. niger *KB (flavan-4-ol [20], 6-hydroxyflavan-4-ol [20], and 7-hydroxyflavan-4-ol [19]) (Table 1). This comparison indicates 2,4-*cis* configuration of 7-methoxyflavan-4-ol (**2**). The proof of this configuration with the help of X-ray analysis was given in our earlier works [19,20]. Observed values of the coupling constants: *J*_2,3eq_ = 2.0 Hz and *J*_2,3ax_ = 11.4 Hz show that the phenyl group is in a pseudoequatorial orientation, whereas *J*_4,3eq_ = 6.3 Hz and *J*_4,3ax_ = 10.2 Hz confirm a pseudoequatorial position of the hydroxyl group at C-4. Protons H-2 and H-4 are in pseudoaxial positions.

**Table 1:** Selected ^1^H-NMR data for 2,4-*cis*-flavan-4-ol [20], 2,4-cis-7-hydroxyflavan-4-ol [19], 2,4-*cis*-6-hydroxyflavan-4-ol [20] and 2,4-cis-7-methoxyflavan-4-ol (**2**).

Compound	δ H-2	δ H-4	δ H-3_ax_	δ H-3_eq_	*J*_3ax-3eq_	*J*_2-3ax_	*J *_2-3eq_	*J *_4-3ax_	*J*_4-3eq_
2,4-*cis*-Flavan-4-ol	5.17	5.08	2.13	2.51	13.1	11.6	1.8	10.6	6.3
2,4-*cis*-7-Hydroxyflavan- 4-ol	5.15	5.00	2.09	2.49	13.2	11.4	1.8	9.7	6.2
2,4-*cis*-6-Hydroxyflavan-4-ol	5.09	4.98	1.98	2.35	12.9	11.9	1.9	10.8	6.4
2,4-*cis*-7-Methoxyflavan-4-ol	5.17	5.05	2.13	2.52	13.2	11.4	2.0	10.2	6.3

**Scheme 2:**
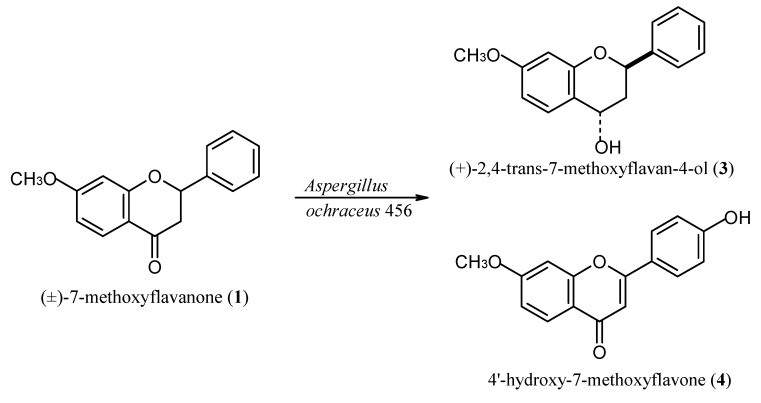
*A. ochraceus *456 catalysed transformation of (±)-7-methoxyflavanone (**1**).

The enzymatic system of *A. ochraceus *456 catalysed transformation of (±)-7-methoxyflavanone (**1**) into two products: (+)-2,4-trans-7-methoxyflavan-4-ol (**3**) and 4'-hydroxy-7-methoxyflavone (**4**) (Scheme 2). After 9 days of biotransformation (+)-2,4-*trans*-7-methoxyflavan-4-ol (**3**) (4.9% yield, 4.9 mg/100 mg of the substrate), and 4'-hydroxy-7-methoxyflavone (**4**) (2.3% yield, 2.3 mg/100 mg of the substrate) were isolated from the reaction mixture.

When monitoring the reaction progress by means of TLC and HPLC we noticed that the amount of product **4**, which is visible starting from the third day of the reaction, was increasing slightly. The reduction product: (+)-2,4-*trans*-7-methoxyflavan-4-ol (**3**) appears in the sixth day, however in the ninth day of the process its amount is higher than the amount of the independently formed 4'-hydroxy-7-methoxyflavone (**4**) (Table 2).

**Table 2:** Biotransformation of 7-methoxyflavanone (**1**), yield (%) of products: 2,4-*trans*-7-methoxyflavan-4-ol (**3**) and 4'-hydroxy-7-methoxyflavone (**4**), according to HPLC (screening tests).

Microorganism	Time of Incubation (days)	Biotransformation products (%)		Unreacted substrate
		4	3	(%)
*Aspergillus ochraceus *456	1	0	0	82.0
	3	2.1	0	35.7
	6	2.3	3.7	18.7
	9	2.5	5.3	9.1

From the first day of the reaction we have observed a considerable decrease in the amount of the substrate **1**, which was not proportional to the amount of products formed (Table 2). These, along with low yield of products, indicate that both 7-methoxyflavanone (**1**) and products **3 **and **4 **may undergo degradation in the culture of *A. ochraceus *456.

In the ^1^H-NMR spectrum of **3 **we can see a multiplet at δ = 5.07 ppm, integrating for one proton, which does not occur in the spectrum of substrate **1 **and which corresponds to H-4. Whereas, in the ^13^C-NMR there is a change in the chemical shift of C-4 from δ = 192.2 ppm for 7-methoxyflavanone (**1**) to δ = 65.6 ppm for 2,4-*trans*-7-methoxyflavan-4-ol (**3**), which is typical for the reaction of reduction. The configuration of 2,4-*trans*-7-methoxyflavan-4-ol (**3**) was confirmed by comparing the ^1^H-NMR spectrum of product **3** with the described earlier spectrum of another carbonyl group reduction product—2,4-*cis*-7-methoxyflavan-4-ol (**2**) (Table 3).

**Table 3:** Selected ^1^H-NMR data for (±)-2,4-*cis*-7-methoxyflavan-4-ol (**2**) and (+)-2,4-*trans*-7-methoxyflavan-4-ol (**3**).

Compound	δ H-2	δ H-4	δ H-3_ax_	δ H-3_eq_	*J*_3ax-3eq_	*J*_2-3ax_	*J*_2-3eq_	*J*_4-3ax_	*J*_4-3eq_
(±)-2,4-*cis*-7-Methoxy- flavan-4-ol (**2**)	5.17	5.05	2.13	2.52	13.2	11.4	2.0	10.2	6.3
(+)-2,4-*trans*-7-Methoxy- flavan-4-ol (**3**)	5.17	5.07	2.95	2.52	13.2	11.5	1.8	3.2	6.2

The observed values of the coupling constants: *J*_2,3ax_ = 11.5 Hz, *J*_2,3eq_ = 1.8 Hz, *J*_4,3ax_ = 3.2 Hz, and *J*_4,3eq_ = 6.2 Hz suggest a pseudoequatorial orientation of the phenyl group and a pseudoaxial position of the hydroxyl group at C-4. Confirmation of the 2,4-*trans* configuration by means of X-ray analysis was described in our earlier papers [19,20].

For the obtained 2,4-*trans*-7-methoxyflavan-4-ol (**3**) the measured specific optical rotation was [α]^20^_546_= +2.57 (c = 0.7, CH_3_OH), and the enantiomeric excess (by HPLC, chiral column) ee = 30%. For the unreacted substrate (**1**) isolated from the reaction mixture the data was as follows: [α]^20^_546_ = +6.28 (c = 2.1, CH_3_OH) and ee = 24%.

The structure of the independently formed 4'-hydroxy-7-methoxyflavone (**4**) was established by means of ^1^H NMR and ^13^C-NMR. Hydroxylation at C 4' in ring B is proved by two doublets integrating each for 2 H at δ = 6.95 ppm (*J* = 8.6 Hz) and δ = 7.81 ppm (*J* = 8.7 Hz), corresponding to H-3', H-5' and H-2', H-6', respectively. In the ^13^C-NMR we observe a change in C-4' chemical shift from δ = 128.8 ppm for substrate (**1**) to δ = 160.5 ppm for product **4**. In the ^1^H-NMR spectrum of 7-methoxyflavone (**1**) the signal of H-2 appears at δ = 5.47 ppm as a doublet of doublets (*J* = 13.3 Hz and *J *= 2.8 Hz), the signal of H-3_ax_ at δ = 3.04 ppm is a doublet of doublets (*J* = 16.9 Hz and *J* = 13.3 Hz) and the signal of H-3_eq_ at δ = 2.84 ppm is also a doublet of doublets (*J* = 16.9 Hz and *J* = 2.9 Hz). In the ^1^H-NMR of 4'-hydroxy-7-methoxyflavone (**4**) a one proton singlet is visible at δ = 5.69 ppm, attributed to H-3, whereas the signal of H-2 disappears, which confirm the presence of a double bond between C-2 and C-3 in ring C. Additionally, in the ^13^C-NMR the signals of C-2 and C-3 were moved from δ = 80.2 ppm and δ = 44.3 ppm for the substrate (**1**) to δ = 162.8 ppm and δ = 104.0 ppm for the dehydrogenation product **4**.

**Scheme 3:**
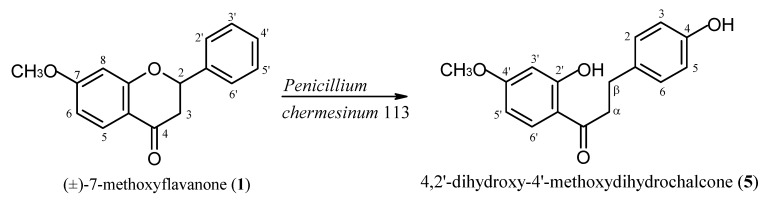
Transformation of (±)-7-methoxyflavanone (**1**) by the strain *P. chermesinum *113.

Transformation of (±)-7-methoxyflavanone (**1**) by the strain *P. chermesinum *113 led to formation of product **5 **of dihydrochalcone structure (Scheme 3).

4,2'-Dihydroxy-4'-methoxydihydrochalcone (**5**) was isolated after 10 days of biotransformation in 15% yield (15 mg/100 mg of the substrate). We expected a 4'-hydroxylation product with the C ring of 7-methoxyflavone intact, which would be analogous to the described earlier hydroxylation of flavanone catalysed by the strain *P. chermesinum *113 [15]. However, when monitoring the reaction progress by TLC and HPLC we did not observe any products other than 4,2'-dihydroxy-4'-methoxydihydrochalcone (**5**).

In the ^1^H-NMR spectrum of product **5** there are two triplets at δ = 2.98 ppm and 3.19 ppm, integrating for two protons each, corresponding to H-β and H-α and typical for a dihydrochalcone structure. Opening of the ring C is additionally proved by the presence of the one proton singlet at δ = 12.6 ppm, which is attributed to 2'-OH. Substitution at C-4 is confirmed by two dublets at δ = 6.77 ppm and δ = 7.11 ppm of identical coupling constants (*J *= 8.5 Hz) and integrating for 2 protons each. The 4-OH hydroxyl proton is visible as a singlet at δ = 5.38 ppm.

In the UV spectra of the biotransformations products the strongest bathochromic shift in the absorption maxima was observed for 4,2'-dihydroxy-4'-methoxydihydrochalcone (**5**): Δλ_max_ = 19 nm (1st band), Δλ_max_ = 9 nm (2nd band), Δλ_max_ = 15 nm (3rd band) (Table 4).

**Table 4:** UV absorption of 7-methoxyflavanone (**1**) and its biotransformation products (**2**-**5**).

Compound	1st band		2nd band		3rd band	
	λ_max_ [nm]	log ε	λ_max_ [nm]	log ε	λ_max_ [nm]	log ε
7-Methoxyflavanone (**1**)	235	4.18	273	4.19	310	3.89
2,4-*cis*-7-Methoxyflavan-4-ol (**2**)	229	3.89	282	3.85	-	-
2,4-*trans*-7-Methoxyflavan-4-ol (**3**)	234	3.81	281	3.87	-	-
4’-Hydroxy-7-methoxyflavone (**4**)	234	4.37	276	4.58	309	4.29
4,2’-Dihydroxy-4’-methoxydihydrochalcone (**5**)	254	4.24	282	4.43	325	4.13

In the case of product **5** a considerable increase in molar absorption coefficient value (ε) compared to substrate **1** was also observed. The highest values of absorption coefficient among the products of 7-methoxyflavanone transformation were measured for 4′-hydroxy-7-methoxyflavone (**4**). Reduction of the carbonyl group of **1** resulted in a decrease in absorption coefficient values in the case of 2,4-*cis* and 2,4*-trans* 7-methoxyflavan-4-ol (**2** and **3**) (Table 4).

The IC_50_ values (antiradical activity) of the substrate and the biotransformation products were determined spectrophotometrically on the basis of graphs: DPPH radical reduction as a function of concentration of a tested compound [16]. IC_50_ means the concentration of an antioxidant (flavonoid) that reduces the initial concentration of DPPH by 50%. The measured IC_50_ values are presented in Table 5.

**Table 5:** The IC_50_ values of the 7-methoxyflavanone (**1**) and the biotransformation products.

Substrate	Product	IC_50_* (± SD) [μM]
7-Methoxyflavanone (**1**)		9.50 (± 0.03)
	4’-Hydroxy-7-methoxyflavone (**4**)	7.66 (± 0.05)
	4,2’-Dihydroxy-4’-methoxydihydrochalcone (**5**)	7.75 (± 0.03)
	2,4-*cis*-7-Methoxyflavan-4-ol (**2**)	8.20 (± 0.06)
	2,4-*trans*-7-Methoxyflavan-4-ol (**3**)	8.42 (± 0.06)

* Mean values of IC_50_ calculated as an average of at least three measurements

Among the products of the microbial transformation of 7-methoxyflavanone the highest antioxidant activity was observed for 4'-hydroxy-7-methoxyflavone (**4**) (IC_50_ = 7.66) and 4,2'-dihydroxy-4'-methoxydihydrochalcone (**5**) (IC_50_ = 7.75). This is due to the microbial introduction of a hydroxyl group at 4' position in ring B. The two reduction products: 2,4-*cis* (**2**) and 2,4*-trans* 7-methoxyflavan-4-ol (**3**) have also higher antioxidant properties than the substrate (**1**). Comparison of the IC_50_ values of **2** and **3 **indicates that 2,4-*cis*-7-methoxyflavan-4-ol (**2**) is a better antioxidant (IC_50_ = 8.20) (Table 5), which shows that stereochemistry of a compound may have an influence on its antioxidant activity. Our earlier research on antioxidant activity of products of biotransformations of flavanone, its monosubstituted derivatives (among them 7-methoxyflavanone) and naringenin allowed us to draw more detailed conclusions concerning the relationship between flavonoid structure and antioxidant activity [16].

## 3. Experimental

### 3.1. Analysis

The course of microbial transformation was monitored by TLC (SiO_2_, DC Alufolien Kieselgel 60 F_254_, Merck, Darmstadt, Germany). Chromatograms were developed using the following developing systems: hexane-ethyl acetate (7:3), dichloromethane-ethyl acetate (1:1), toluene-diethyl ether (4:1). Column chromatography (SiO_2_, Kieselgel 60, 230–400 mesh, 40–63 μm, Merck) was performed using the same eluents. ^1^H-NMR and ^13^C-NMR spectra were recorded with a Bruker Avance DRX 300 spectrometer. IR spectra were determined with a Mattson IR 300 Thermo Nicolet spectrometer. Mass spectra were obtained using high-resolution electrospray ionization (ESI^+^-MS) (Waters LCT Premier XE mass spectrometer).

HPLC analyses were performed with a Waters 2690 instrument equipped with Waters 996 photodiode array detector, using ODS 2 column (4.6 × 250 mm, Waters) and a Guard-Pak Inserts μBondapak C18 pre-column. Separation conditions were as follows: gradient elution, using 80% of acetonitrile in 4.5% formic acid solution (eluent A) and 4.5% formic acid (eluent B); flow, 1 mL/min; detection wavelength 280 nm; program: 0–7 min, 10% A 90% B; 7–10 min, 50% A 50% B; 10–13 min, 60% A 40% B; 13–15 min, 70% A 30% B; 15–20 min 80% A 20% B; 20–30 min 90% A 10% B; 30–40 min, 100% A. Melting points were determined with a Boetius apparatus (Kofler block). Antioxidant properties were measured with a Cintra 20 spectrometer (GBC, Melbourne, Australia). 

### 3.2. Materials

The racemic substrate for biotransformation, 7-methoxyflavanone (**1**), was purchased from Aldrich (Poznań, Poland). C_16_H_14_O_3_; Melting point 89–91 °C; Full description of the ^1^H-NMR and ^13^C-NMR spectra can be found in our previous paper [20].

#### Microorganisms

The wild strain *A. niger *KB was obtained from the collection of the Department of Biotechnology and Food Microbiology of Wrocław University of Environmental and Life Sciences (Poland). The microorganism was maintained on potato slants (sterilized piece of potato) at 5 °C.

The wild strains *A. ochraceus *456 and *P. chermesinum *113 were obtained from the collection of the Department of Chemistry of Wrocław University of Environmental and Life Sciences (Poland). The microorganisms were maintained on agar slants at 5 °C.

### 3.3. Biotransformations

#### 3.3.1. Screening Procedure

Cultivation media consisted of 3% glucose (The Industrial and Trading Enterprise “Stanlab” Co. Ltd., Lublin, Poland) and 1% peptobac (BTL sp. z o.o., Warszawa, Poland) in water. The microorganisms were transferred from the slants to 500 mL Erlenmayer flasks, each containing 200 mL of the medium. Preincubation was performed at 25 °C for 24–48 h. Then portions of 1 mL of the culture solution were transferred to inoculate 500 mL flasks, each containing 200 mL of the medium. After cultivation at 25 °C for 24 h on a rotary shaker, 10 mg of a substrate, dissolved in 0.5 mL of THF, was added to the grown culture. Control cultivation with no substrate was also performed. After 1, 3, 6 and 9 days of incubation under the above conditions, portions of 5 mL of the transformation mixture were withdrawn and extracted with ethyl acetate (3 × 3 mL). The extracts were dried over MgSO_4_ (5 min), concentrated *in vacuo* and analyzed by TLC. Quantitative analyses of the mixtures were performed by means of HPLC. Calibration curves for quantitative analyses were prepared using isolated and purified biotransformation products as standards.

#### 3.3.2. Preparative Biotransformation

Portions of 1 mL of the preincubation culture solution were used to inoculate three 2000 mL flasks, each containing 500 mL of the cultivation medium. The cultures were incubated at 25 °C for 48 h on a rotary shaker. Then 50 mg of the substrate dissolved in 2.5 mL of THF was added to each flask (100 mg of the substrate per 1 L of the cultivation mixture). After 9 or 10 days of incubation the mixtures were extracted with ethyl acetate (3 × 200 mL), dried (MgSO_4_) and concentrated *in vacuo*. The transformation products were separated by column chromatography. Pure products were identified by means of spectral analyses (TLC, ^1^H-NMR, ^13^C-NMR, IR).

The physicochemical and spectrometric data of the products were as follows:

*(±)-2,4-cis-7-Methoxyflavan-4-ol *(**2**) [21]. C_16_H_16_O_3_; Melting point 116–118 °C; 76% yield; purity 98% (HPLC); [α]^23^_546_ = 0, (c = 1.0, CH_3_OH); ^1^H-NMR (CDCl_3_) δ: 2.13 (1H, ddd, *J*_3ax,3eq_ = 13.2 Hz, *J*_3ax,2 _= 11.4 Hz, *J*_3ax,4 _= 10.2 Hz, H-3_ax_), 2.52 (1H, ddd, *J*_3eq,3ax_ = 13.2 Hz, *J*_3eq,4_= 6.3 Hz, *J*_3eq,2_ = 2.0 Hz, H-3_eq_), 3.79 (3H, s, 7-OCH_3_), 5.05 (1H, broad s, H-4), 5.17 (1H, dd, *J*_2,3ax_ = 11.4 Hz, *J*_2,3eq_ = 2.0 Hz, H-2), 6.45 (1H, d, *J*_8,6_ = 2.5 Hz, H-8), 6.59 (1H, dd, *J*_6,5 _= 8.6 Hz, *J*_6,8 _= 2.5 Hz, H-6), 7.36 (1H, d, *J*_5,6_ = 8.6, H-5), and 7.34–7.45 (5H, m, B-ring protons); ^13^C-NMR (CDCl_3_) δ: 40.2 (C-3), 55.3 (OCH_3_), 65.6 (C-4), 77.1 (C-2), 101.1 (C-8), 108.1 (C-6), 118.2 (C-10), 126.0 (C-2', C-6'), 127.8 (C-4'), 128.1 (C-5), 128.6 (C-3′, C-5′), 140.4 (C-1'), 155.5 (C-9), and 160.3 (C-7); IR (CH_2_Cl_2_, ν_max_, cm^−1^): 3225 (O-H, stretch), 1619 (C-C, stretch, aromatic), and 1590 (C-C, stretch, aromatic); HRESI-MS [M+H^+^] (calculated/found) (*m/z* 257.0990/257.0979).

*(+)-2,4-trans-7-Methoxyflavan-4-ol *(**3**). C_16_H_16_O_3_; Melting point 115–117 °C; 4.9% yield; purity 97% (HPLC); [α]^20^_546_ = +2.57, (c = 0.7, CH_3_OH), *ee* = 30%; ^1^H-NMR (CDCl_3_) δ: 2.52 (1H, ddd, *J*_3eq,3ax_ = 13.2 Hz, *J*_3eq,4_ = 6.2 Hz, *J*_3eq,2_ = 1.9 Hz, H-3_eq_), 2.95 (1H, ddd, *J*_3ax,3eq_ = 13.1 Hz, *J*_3ax,2_ = 12.0 Hz, *J*_3ax,4_ = 3.2 Hz, H-3_ax_), 5.07 (1H, m, H-4), 5.17 (1H, dd, *J*_2,3ax_ = 11.5 Hz, *J*_2,3eq_ = 1.8 Hz, H-2), 6.45 (1H, d, *J*_8,6_ = 2.5 Hz, H-8), 6.58 (1H, dd, *J*_6,5_ = 8.4 Hz, *J*_6,8_ = 2.5 Hz, H-6), 7.42 (1H, d, *J*_5,6_ = 8.5, H-5), and 7.34–7.47 (5H, m, B-ring protons); ^13^C-NMR (CDCl_3_) δ: 40.0 (C-3), 55.2 (7-OCH_3_), 65.6 (C-4), 77.2 (C-2), 101.2 (C-8), 108.1 (C-6), 118.0 (C-10), 126.0 (C-2', C-6'), 127.9 (C-4'), 128.2 (C-5), 128.7 (C-3', C-5'), 140.1 (C-1'), 155.0 (C-9), and 160.1 (C-7); IR (CH_2_Cl_2_, ν_max_, cm^−1^): 3230 (O-H, stretch), 1619 (C-C, stretch, aromatic) and 1595 (C-C, stretch, aromatic); HRESI-MS [M+H^+^] (calculated/found) (*m/z* 257.0990/257.0977).

*4*'*-Hydroxy-7-methoxyflavone *(**4**) [22]. C_16_H_12_O_4_; Melting point 195–197 °C; 2.3% yield; purity 98% (HPLC); ^1^H-NMR (CDCl_3_) δ: 3.78 (3H, s, 7-OCH_3_), 5.69 (1H, s, H-3), 6.51 (1H, d, *J*_8,6_ = 2.5 Hz, H-8), 6.57 (1H, dd, *J*_6,5_ = 8.4 Hz, *J*_6,8_ = 2.5 Hz, H-6), 6.95 (2H, d, *J* = 8.6 Hz, H-3', H-5'), 7.42 (1H, d, *J*_5,6_ = 8.3 Hz, H-5), 7.81 (2H, d, *J* = 8.7 Hz, H-2', H-6'), and 9.88 (1H, s, 4'-OH); ^13^C-NMR (CDCl_3_) δ: 55.4 (7-OCH_3_), 101.4 (C-8), 104.0 (C-3), 108.4 (C-6), 115.9 (C-3', C-5'), 122.0 (C-1'), 126.3 (C-10), 128.1 (C-5), 128.6 (C-2', C-6'), 160.5 (C-4'), 161.2 (C-9), 162.8 (C-2 ), 168.6 (C-7), and 174.6 (C-4); IR (KBr, ν_max_, cm^−1^): 3432 (O-H, stretch), 1670 (C = O, stretch), 1405 (C-C, stretch, aromatic) and 774 (C-H, bending, aromatic); HRESI-MS [M+H^+^] (calculated/found) (*m/z* 269.0965/269.0960).

*4,2*'*-Dihydroxy-4*'*-methoxydihydrochalcone *(**5**) [23]. C_16_H_16_O_4_; Melting point 121–123 °C; 15% yield; purity 99% (HPLC); ^1^H-NMR (CDCl_3_) δ: 2.98 (2H, t, *J* = 7.5 Hz, H-β), 3.19 (2H, t, *J* = 7.8 Hz, H-α), 3.83 (3H, s, 4'-OCH_3_), 5.38 (1H, s, 4-OH), 6.40 (1H, d, *J* = 2.5 Hz, H-3’), 6.42 (1H, dd, *J* = 9.0 Hz, *J* = 2.4 Hz, H-5'), 6.77 (2H, d, *J* = 8.5, H-3, H-5), 7.11 (2H, d, *J* = 8.5, H-2, H-6), 7.63 (1H, d, *J* = 9.0, H-6'), and 12.60 (1H, s, 2'-OH); ^13^C-NMR (CDCl_3_) δ: 29.9 (C-β), 40.1 (C-α), 55.6 (-OCH_3_), 101.2 (C-3'), 107.5 (C-5'), 114.0 (C-1'), 115.7 (C-3, C-5), 129.5 (C-2, C-6), 131.3 (C-6'), 133.0 (C-1), 155.1 (C-4), 166.1 (C-2'), 166.7 (C-4'), and 191.5 (C=O); IR (CH_2_Cl_2_, ν_max_, cm^−1^): 3576 (O-H, stretch), 1706 (C = O, stretch), 1650 (C-C, stretch, aromatic) and 1480 (C-C, stretch, aromatic); HRESI-MS [M+H^+^] (calculated/found) (*m/z* 273.1111/273.1105).

### 3.4. Measurement of Antioxidant Properties of the Substrate and the Products

A methanolic solution of DPPH (1,1-diphenyl-2-picryl-hydrazyl) with an absorbance of about 1.00, was mixed with a proper amount of a tested flavonoid **1**–**5**. After 20 min, disappearance of absorbance at 520 nm was measured. The initial concentration of DPPH was determined by means of calibration curve. The IC_50_ value (antiradical activity) was determined graphically—DPPH radical reduction (expressed in %) as a function of concentration of the tested compound. IC_50_ means concentration of the antioxidant that reduces the initial concentration of DPPH by half. 

## 4. Conclusions

The study on *A. niger* KB described in this article and in our previous papers prove that this strain is a good catalyst for carbonyl group reduction in flavanone and its monosubstituted derivatives [19,20]. The strain *P. *chermesinum 113 performs mainly reactions of hydroxylation in ring B, which are often accompanied with the ether bond cleavage in ring C, leading to dihydrochalcone structure [15].

The biotransformations of 7-methoxyflavanone did not afford any enantiomerically pure product. Enantiomeric excess of 2,4-*trans*-7-methoxyflavan-4-ol obtained in the reaction catalysed by *A. ochraceus *456 did not exceed 30%.

All the products of biotransformations of 7-methoxyflavanone have higher antioxidant properties than the substrate. 
